# Supplementary material for: Development of an In-Line Enzyme Reactor Integrated into a Capillary Electrophoresis System
Source: Molecules. 2021 Sep 29;26(19):5902. doi: 10.3390/molecules26195902 (PMC8513095; doi:10.3390/molecules26195902)
Supplement: Supplementary file 1 [file molecules-26-05902-s001.zip › molecules-1393930-supplementary.pdf]

Development of an in-line enzyme reactor integrated into a capillary electrophoresis system

Cynthia Nagy, Ruben Szabo, Attila Gaspar*

Department of Inorganic and Analytical Chemistry, University of Debrecen, Egyetem ter 1., Debrecen 4032, Hungary

*Corresponding author

E-mail: gaspar@science.unideb.hu

Tel: +36-30-2792889, Fax: +36-52-518660

Supplementary Materials

Table S1. Detailed injection parameters for cases **1-7** of Figure 1.

	inlet vial	action	time	pressure
**1**	sample	pressure	2 s	50 mbar
	inlet BGE	pressure	10 s	50 mbar
**2**	sample	pressure	2 s	50 mbar
	inlet BGE	pressure	10 s	50 mbar
	inlet BGE	wait	60 s	
**3**	sample	pressure	2 s	50 mbar
	inlet BGE	pressure	3 s	50 mbar
	inlet BGE	wait	60 s	
	inlet BGE	pressure	7 s	50 mbar

	inlet vial	action	time	pressure
**4**	sample	pressure	2 s	50 mbar
	inlet BGE	pressure	8 s	50 mbar
**5**	sample	pressure	2 s	50 mbar
	inlet BGE	pressure	30 s	15 mbar
**6**	sample	pressure	2 s	50 mbar
	inlet BGE	pressure	4×2 s	50 mbar
**7**	sample	pressure	2 s	50 mbar
	inlet BGE	pressure	2 s	50 mbar
	inlet BGE	wait	60 s	
	inlet BGE	pressure	6 s	50 mbar

Table S2. Preconditioning and injecting parameters for the generation of the immobilized trypsin layer in the case of CE-DAD measurements.

	inlet vial	outlet vial	action	time	pressure	
preconditioning	washing BGE	empty vial	flush	60 s	1 bar	
	trypsin solution	washing BGE	pressure	8 s	50 mbar	
	trypsin solution	washing BGE	wait	60 s		×2
	trypsin solution	washing BGE	pressure	12 s	-50 mbar	
	empty vial	washing BGE	pressure	120 s	-50 mbar	
	inlet BGE	outlet BGE	flush	60 s	1 bar	
injecting	sample	outlet BGE	pressure	2 s	50 mbar	
	inlet BGE	outlet BGE	pressure	8 s	50 mbar	

Table S3. Preconditionings and injecting parameters for the CE-MS measurements.

	inlet vial	action	time	pressure	
preconditioning	washing BGE	flush	180 s	1 bar	
	trypsin solution	pressure	40 s	50 mbar	×3
	trypsin solution	wait	60 s		
	trypsin solution	pressure	60 s	-50 mbar	
	empty vial	pressure	120 s	-50 mbar	
	inlet BGE	flush	60 s	1 bar	
injecting	sample	pressure	6 s	50 mbar	
	inlet BGE	pressure	60 s	15 mbar	
	inlet BGE	wait	300 s		
	inlet BGE	pressure	60 s	15 mbar	

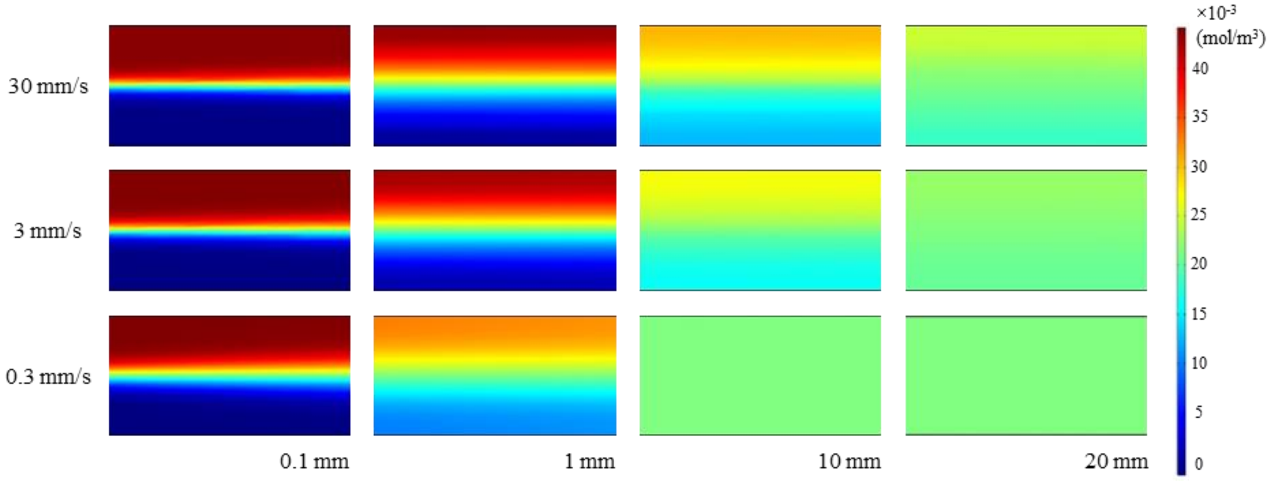

Figure S1. COMSOL simulations showing the effect of linear flow velocity on diffusion in a 50 µm ID capillary. The three rows correspond to three different linear flow velocities of increasing orders of magnitude. The simulations show the concentration (mol/m^3) distribution of albumin across the capillary. Water and albumin were introduced at the inlet at 1:1 ratio. Four channel segments were magnified for better visibility. Values at the bottom mark the distance from entry point (inlet end of the capillary). (L: 2 cm; $D_{albumin}$ = $6.1 \cdot 10^{-11}$ m^2/s).

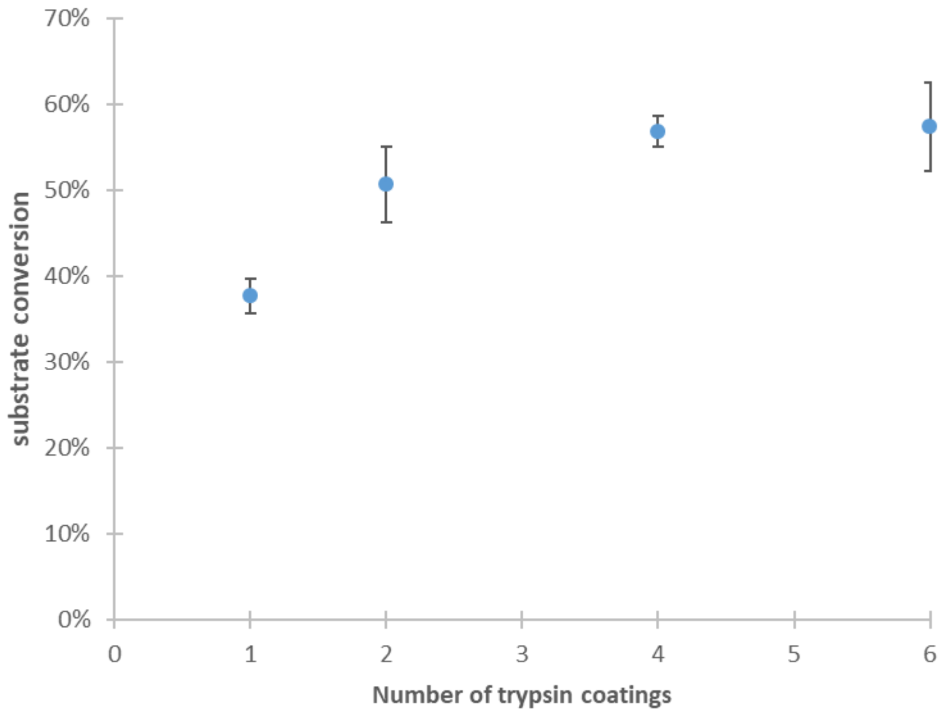

Figure S2. The effect of the number of trypsin coatings on reactor activity. Conditions were the same as in Figure 2.

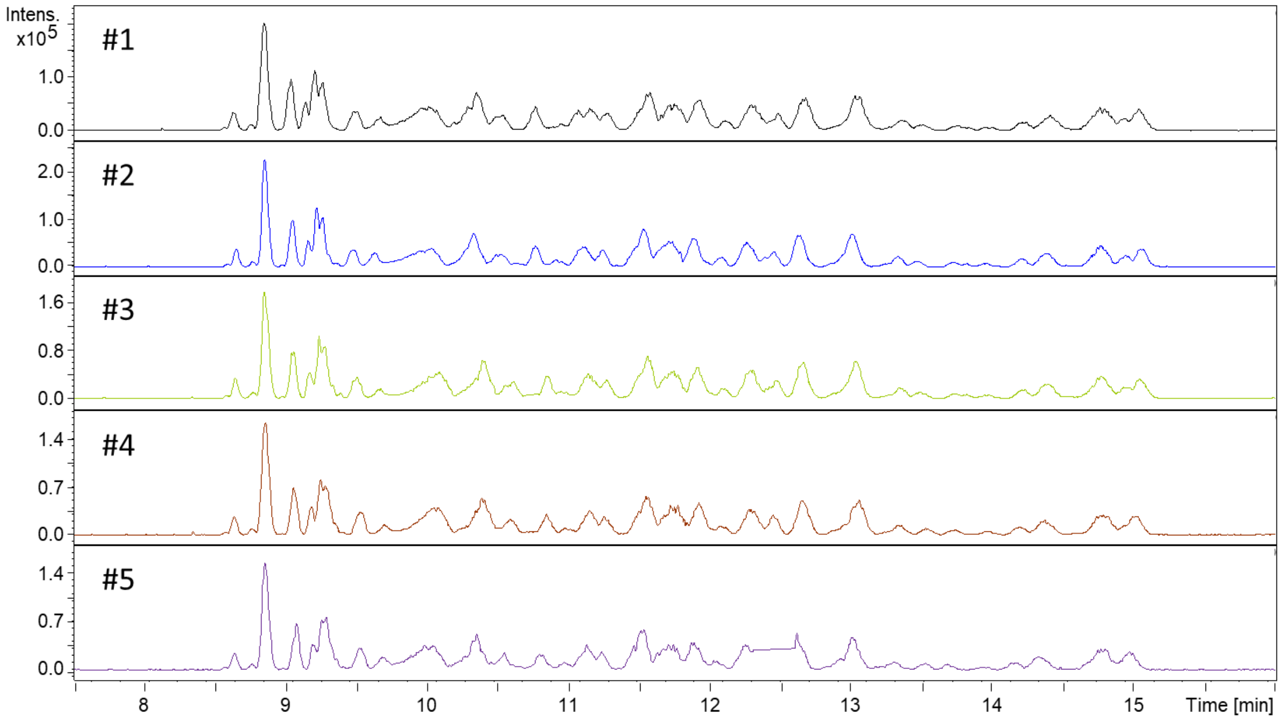

Figure S3. Repeatability of CZE-MS measurements after in-solution digestion of human serum albumin. Conditions were the same as in Figure 3.E, the trypsin layer generation (rows with gray background in Table S2) was omitted.

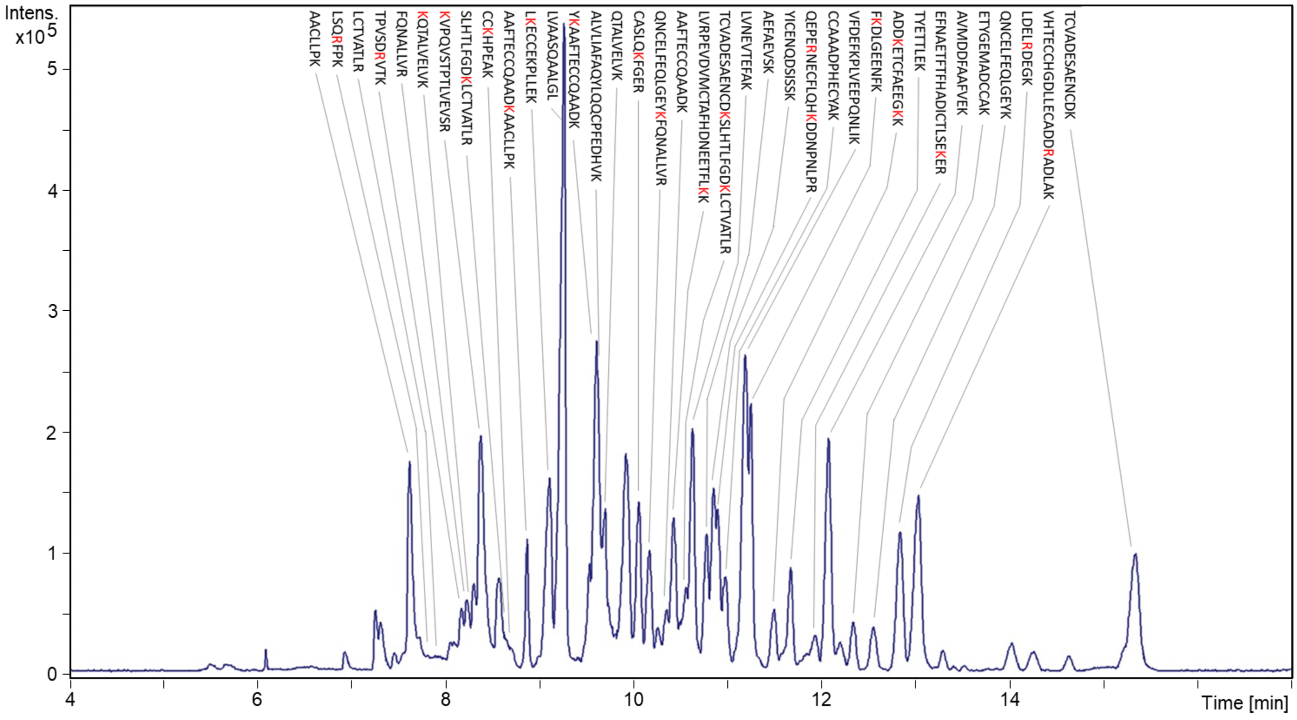

Figure S4. CZE-MS electropherogram obtained after in-line digestion of human serum albumin with a 7.5 minute contact time. The peptide sequences correponding to the peaks are shown and the missed cleavages are highlighted in red. Conditions were the same as in Figure 3. E.
